# HLA-E Binding Peptide as a Potential Therapeutic Candidate for High-Risk Multiple Myeloma

**DOI:** 10.3389/fonc.2021.670673

**Published:** 2021-06-09

**Authors:** Ying Yang, Zhuogang Liu, Hongtao Wang, Guojun Zhang

**Affiliations:** Department of Hematology, Shengjing Hospital of China Medical University, Shenyang, China

**Keywords:** HLA-E, high risk, multiple myeloma, clinical outcomes, target-binding peptide

## Abstract

Human leukocyte antigen-E (HLA-E) has been putatively associated with the pathogenesis of multiple myeloma (MM). Our study first showed that HLA-E was differentially expressed on MM and normal plasma cells (39.27 ± 27.01 and 11.28 ± 0.79, respectively). Based on the median value of HLA-E expression, we further stratified MM patients into high and low-expression groups, and then found high expression of HLA-E was correlated with advanced ISS stage (p = 0.025) and high-risk cytogenetics risk stratification (p = 0.000) by the Pearson Chi-square test, suggesting that HLA-E could be considered as a biomarker for high-risk MM. Furthermore, peptide 3 (P3) from our previous study was confirmed to possess a high affinity to HLA-E positive MM cells. Taken together, HLA-E could be considered as a new marker and candidate treatment target for MM, while peptide P3 may act as a potential treatment choice for targeting MM cells.

## Introduction

Multiple myeloma (MM) is a common malignant hematological disease originating from plasma cells (1), and its prognosis has remarkably improved as treatment regimens have evolved into currently more popularized immunotherapies (2–6). As one type of immunotherapeutic regimens, monoclonal antibodies, such as Daratumumab (CD38 antibody), has exhibited significant treatment efficacy in both patients with MM and with relapsed/refractory MM (RRMM) (7). However, a certain percentage of MM patients have been profiled as high-risk for RRMM with much shorter progression-free survival (PFS) and overall survival (OS). Therefore, early identification of myeloma patients with a high-risk of refractory or relapse and development of targeted treatment regimen remain the priorities in the study of MM.

HLA-E is a non-classical major histocompatibility complex (MHC) class I molecule characterized by lower polymorphism, which plays a critical role in the immune response by both inhibiting and activating the function of natural killer (NK) cells (8). Studies have shown that HLA-E expression correlates with worse progression-free survival in newly diagnosed, treatment-naïve MM patients. Based on a bioinformatics analysis in our previous work, we suggest HLA-E as a potential therapeutic target for the treatment of MM (9) and designed peptides to bind HLA-E by analyzing its interaction with CD94/NKG2A. Thereafter, a peptide library was built upon the strategy of randomly replacing non-key amino acids to enhance the affinity of peptides (10), in which the top three peptides were subjected to molecular docking analysis. Subsequently, a peptide designated as P3 (NALDEYCEDKNR) was found to have the highest affinity for HLA-E, indicating that P3 could be considered as a potential inhibitor to specifically target MM cells (9). Thus, the present study aims to continue our investigation on the clinical meaning of HLA-E expression in MM patients and further explore whether peptide P3 could target HLA-E positive myeloma cells.

## Materials and Methods

### General Information

This study, which included 30 newly diagnosed multiple myeloma (NDMM) patients from January 1, 2018 to November 31, 2019, was approved by the ethics committee of Shengjing hospital of China Medical University (2020PS215K). Following the diagnoses of MM according to the International Myeloma Working Group (IMWG) guidelines for symptomatic MM (11) and acquiring patients’ consents, all bone marrow samples were collected. All patients were classified according to the staging criteria (12). Patients were excluded from this study if they had histories of any immune deficiency disease, transplantation or other malignant tumor, or previous immunosuppressive therapy. For the purpose of analysis, the baseline data of gender, age, clinical stage, typing, and immunoglobulin heavy chain (IgH) quantity were recorded, while bone marrow from non-malignant patients was collected for use as the control.

### Flow Cytometric (FCM) Analysis

The expressions of HLA-E, CD138, and CD45 were determined by a flow cytometer (FACS Calibur; Becton Dickinson, San Diego, CA, USA) with mouse antihuman fluorescent monoclonal antibodies [fluorescein isothiocyanate (FITC), phycoerythrin (PE), peridinin-chlorophyll-protein (Percp) and allophycocyanin (APC)]. The antibodies were purchased from BD Pharmingen (San Diego, CA, USA). After incubation with antibodies in the dark for 15 min, flow cytometry with CD138++/CD45 was performed on at least 50,000 cells for gating the viable cells. The HLA-E antigen expression was further analyzed with CellQuest software (Becton Dickinson). Target-binding peptides labeled by FITC (peptide M and peptide P3) were synthesized by Chinese Peptide (Hangzhou, China). Then, the affinity of peptides against HLA-E on the collected bone marrow from the MM patients was detected by FCM, and the binding affinity was further analyzed on the positive portion of the target-binding peptide.

### Statistical Analysis

The Mann–Whitney U test was used to compare the difference between non-normal distribution data, while the Pearson chi-square test was employed to compare the correlation between HLA-E expression and clinic-pathologic parameters. MM patients with HLA-E expression were divided into high-expression and low-expression groups based on the mean value. SPSS24.0 (Chicago, IL, USA) and GraphPad PRISM 6.0 (La Jolla, CA, USA) were used for statistical analysis, and *p* < 0.05 was considered statistically significant.

## Results

### General Characteristics

A total of 30 patients diagnosed with MM, according to IMWG guidelines, were evaluated, including 18 males and 12 females with a median age of 65 years (47-83 years). All patients received Bortezomib-based regime as the standard chemotherapy. The general characteristics of these MM patients are summarized in **Table 1**. Additionally, bone marrow from seven patients with a non-malignant hematological disease were selected as a control for the present study. High-risk cytogenetic features of MM patients were detected by fluorescence *in situ* hybridization (FISH).

**Table 1:** General characteristics of the patients with newly diagnosed MM.

Characteristics	NDMM
Gender (male/female)	18/12 (60/40%)
Age (years)	65 (47–83)
Immunoglobin types (n/%)	
*IgG*	12/40%
*IgA*	5/16.7%
*IgD*	1/3.3%
*Light chain*	11/36.7%
*Non-secretory*	1/3.3%
DS staging system (n/%)	
*Stage I*	3/10%
*Stage II*	9/30%
*Stage III A*	12/40%
*Stage III B*	6/20%
ISS staging system (n/%)	
*Stage I*	3/10%
*Stage II*	8/26.7%
*Stage III*	19/63.3%
R-ISS staging system (n/%)	
*Stage I*	2/6.7%
*Stage II*	10/33.3%
*Stage III*	18/60%
Cytogenetic risk factors * (n/%)	
*Standard risk*	21/70%
*High risk*	9/30%

*According to Mayo Clinic mSMART 3.0: Classification of active MM. The genetic abnormalities for high risk of MM include t(4;14); t(14;16); t(14;20); Del 17p; Gain 1q.

### Identification of High Expression of HLA-E Protein on Multiple Myeloma

**Figure 1:**
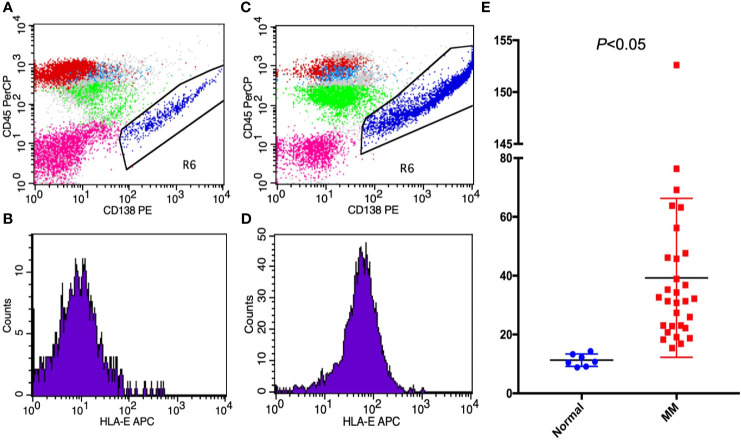
The expressions of **(A, B)** HLA-E on non-malignant hematological patients; **(C, D)** HLA-E in MM patients; and **(E)** HLA-E between MM patients and non-malignant patients presented by mean fluorescence intensity.

HLA-E was detected in 30 MM patients and in 7 non-malignancy control patients by FCM. In MM patients, CD138 and CD38 were strongly positive in abnormal plasma cells, thus indicating that the CD138 antigen can be used to identify MM cells. According to the quantitative analysis of FCM results, the mean fluorescence intensity of HLA-E was 39.27 ± 27.01 (15.4-152.61) in MM cells and 11.28 ± 0.79 (8.82-14.33) in control cells with positive CD138. These results show that HLA-E was highly expressed in MM patients (*p* < 0.05) (**Figure 1**).

### High HLA-E Expressed in Advanced Stage Multiple Myeloma

The relationship between HLA-E expression and age, gender, stage, and cytogenetic risk stratification in the 30 patients with MM was further analyzed. Considering the median expression value of HLA-E protein of 31.77, median age of 65 years old, and R-ISS staging system, the MM patients were stratified into high-expression and low-expression groups, older and younger age groups, and early stage and advanced stage groups, respectively. Furthermore, the early stage was divided into stages I and II, which included 12 patients, and the advanced stage was categorized as stage III with 18 patients. Patients were also divided into two groups based on cytogenetic risk. The Pearson Chi-square test showed that high HLA-E expression was correlated with advanced ISS stage (*p* = 0.025) and high cytogenetic risk (*p* = 0.000) (**Table 2**). Therefore, the high expression of HLA-E in advanced stage, high-risk MM patients may predict poor prognosis, indicating that HLA-E could be considered as a treatment target, especially for high-risk MM patients.

**Table 2:** The relationship between HLA-E and clinical parameters.

Characteristics	Expression of HLA-E		
	Low expression n (%)	High expression n (%)	*p*
Age (years)			
*≤65*	7 (43.8%)	9 (56.2%)	0.464
*>65*	8 (57.1%)	6 (42.9%)	
Gender			
*Male*	8 (44.4%)	10 (55.6%)	0.456
*female*	7 (58.3%)	5 (41.7%)	
R-ISS staging system			
*Early stage*			
*(stage I–II)*	9 (75%)	3 (25%)	**0.025**
*Advanced stage*			
*(stage III)*	6 (33.3%)	12 (66.7%)	
Cytogenetics risk			
*Standard risk*	15 (71.4%)	6 (28.6%)	**0.000**
*High risk*	0 (0%)	9 (100%)	

*The cutoff value of HLA-E was 31.77 based on the median expression value. Bold value means having statistically signiﬁcant.

### Binding Frequency of the Target-Binding Peptides to HLA-E in Multiple Myeloma

**Figure 2:**
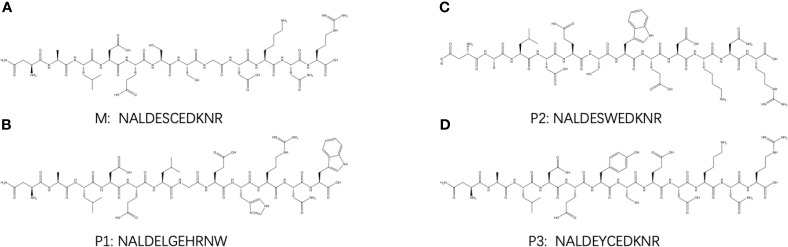
The structure of HLA-E targeted binding peptides: **(A)** M, **(B)** P1, **(C)** P2, and **(D)** P3.

**Figure 3:**
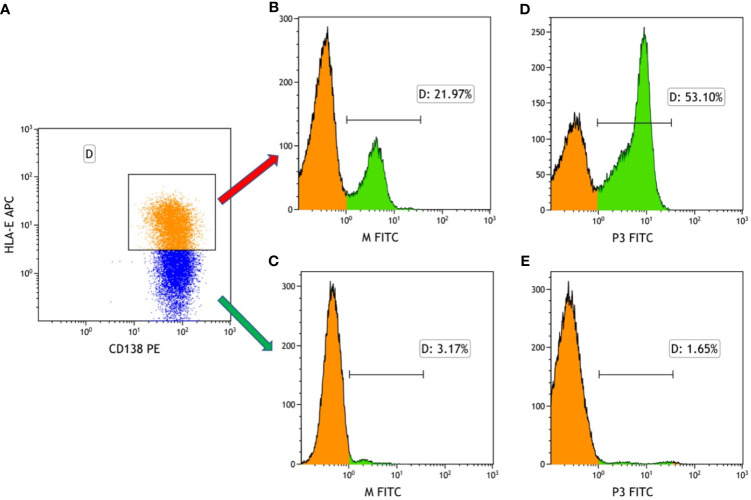
The binding frequency of HLA-E-targeted binding peptides to the bone marrow cells from the patients with NDMM. **(A)** Myeloma cells were divided into HLA-E+ cells and HLA-E- cells. **(B)** The binding affinity of peptide M to HLA-E positive myeloma cells was 21.97%. **(C)** The affinity of peptide M to HLA-E negative myeloma cells was 3.17%. **(D)** The binding affinity of peptide P3 to HLA-E positive myeloma cells was 53.10%. **(E)** The binding affinity of peptide M to HLA-E negative myeloma cells was 1.65%.

As mentioned, the results show that HLA-E was highly expressed on myeloma cells. In our previous work, the Molecular Operating Environment (MOE) software was employed to screen four target-binding peptides, namely M and P1-P3, for their affinity to HLA-E. Furthermore, these four peptides were synthesized and labeled with FITC fluorescein, for which the amino acid sequences are listed in **Figure 2**. The results from our previous work also indicate that P3 specifically binds to HLA-E highly expressed in cell lines with the highest affinity compared to the three other peptides. The purity and molecular weights of these peptides are provided in **Supplementary Material**. As shown in **Figure 3A**, CD138+HLA-E+ and CD138+HLA-E- myeloma cells were cultured with peptide M and P3. It was found that both peptide M and P3 could interact with CD138+HLA-E+ cells but not with CD138+HLA-E- myeloma cells. Specifically, the proportions of FITC-labeled peptide M and P3 on CD138+HLA-E+ cells were 21.97% and 53.1%, respectively (**Figures 3B, D**), but were only 3.17% and 1.65% on CD138+HLA-E- cells (**Figures 3C, E**).

## Discussion

Immune function plays an important role in MM (13), whereby the absolute lymphocyte count (ALC) is related to the prognosis of MM patients (14). Therefore, a high level of ALC in NDMM patients leads to a better prognosis even in the new immunotherapy era. Although HLA-E has been screened as the key membrane antigen in the development of MM by the bioinformatics method (9), the clinical meaning of the different expressions of HLA-E in MM patients remains unknown. In the present study, we found that the HLA-E protein is highly expressed on MM cells and is linked to high-risk MM. Thus, HLA-E could be considered as both a marker of high-risk MM and a targeted candidate in a new treatment regimen for MM patients.

As a non-classical major histocompatibility complex (MHC) molecule, HLA-E can interact with NK cells and T cells (15, 16). It has been suggested that the overexpression of HLA-E on cells could inhibit the immune clearance function (17). On some tumors, including MM, inflammatory cells and senescent cells with highly expressed HLA-E could escape the NK and T-cell immune surveillance and stay alive in the host body (18–21). Herein, we found that HLA-E was expressed much higher on MM cells than normal plasma cells, especially in NDMM patients. While cytogenetic risk factors are typically used to predict the prognosis of MM patients (22), introducing new medication, such as protease inhibitor, immunomodulatory agents, and monoclonal antibodies, could significantly improve prognoses (7, 23). However, some patients still suffer from disease progression despite these new therapies. Therefore, new prognostic markers and novel therapeutic targets should be investigated for identifying the patients with high-risk MM and improving treatment efficacy.

Furthermore, we divided MM patients into two groups based on the median HLE-A expression value of 31.77. On one hand, no statistical difference existed between the different age and sex groups. On the other hand, the group with a high risk of MM and the standard-risk group showed a significant difference in the expression of HLA-E. Comparatively, HLA-E was expressed much higher in the high-risk group than the low-risk one. This indicates that HLA-E could be considered as a marker for predicting whether the patients have high-risk myeloma or not. Besides, HLA-E might be taken as a potential treatment target for MM, especially for high-risk patients. Since HLA-E overexpression could inhibit the immune clearance function of NK cells, we propose that MM cells could be eliminated by either targeting HLA-E or inhibiting the interaction between HLA-E and NKG2A using peptide P3 (15, 24, 25). In our future work, we aim to examine the effect of peptide P3 on recovering the killing function of NK cells by inhibiting the interaction between HLA-E and NKG2A. Yet, if this recovery cannot be done by peptide P3, HLA-E could be considered as a target to find MM cells, and then a peptide drug conjugate could be produced to target MM cells.

Target-binding peptides M and P1-3, which were designed and synthesized in our previous work, could interact with the HLA-E protein in MM cell lines (9). Comparatively, peptide M exhibited the lowest binding affinity with HLA-E, while P3 showed the highest affinity. The present study further verified this finding in the bone marrow of MM patients. The results confirm that peptide P3 could bind to HLA-E positive cells but cannot interact with HLA-E negative cells in bone marrow.

## Conclusions

The results of this study reveal the overexpression of HLA-E on MM cells, especially of high-risk patients, and the high binding frequency of peptide P3 to HLA-E on MM patients *in vitro*. From a therapeutic perspective, HLA-E can be considered as an effective targeting therapy against MM cells, while P3 specifically binds with HLA-E. Consequently, this interrupts the interaction between HLA-E and the inhibitory receptor NKG2A, providing a promising strategy to improve the immune clearance of MM cells.

## Data Availability Statement

The raw data supporting the conclusions of this article will be made available by the authors, without undue reservation.

## Ethics Statement

The studies involving human participants were reviewed and approved by ethics committee in Shengjing hospital, 2020PS215K. The patients/participants provided their written informed consent to participate in this study. Written informed consent was obtained from the individual(s) for the publication of any potentially identifiable images or data included in this article.

## Author Contributions

HW and YY conceived and designed the study. YY and GZ performed the experiment and collected the data. GZ and ZL performed the data analysis and statistical analysis. YY, HW, and GZ revised the manuscript. All authors contributed to the article and approved the submitted version.

## Funding

This work was supported by the 345 Talent Project in Shengjing Hospital of China Medical University.

## Conflict of Interest

The authors declare that the research was conducted in the absence of any commercial or financial relationships that could be construed as a potential conflict of interest.
