# Supplementary figures and images for: HLA-E Binding Peptide as a Potential Therapeutic Candidate for High-Risk Multiple Myeloma
Source: Front Oncol. 2021 Jun 9;11:670673. doi: 10.3389/fonc.2021.670673 (PMC8219970; doi:10.3389/fonc.2021.670673)

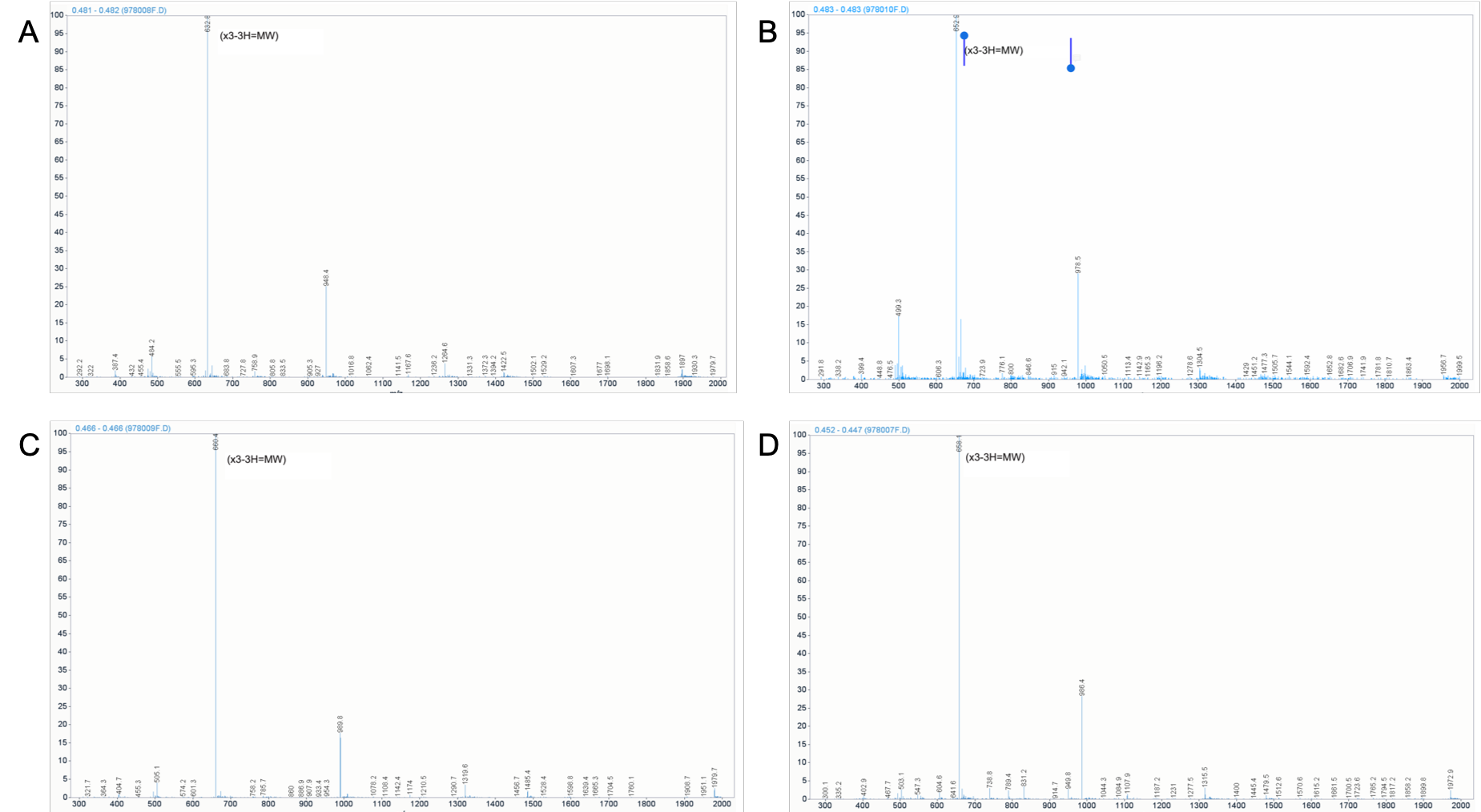

Supplement: Supplementary file 1 [file Image_1.tiff]

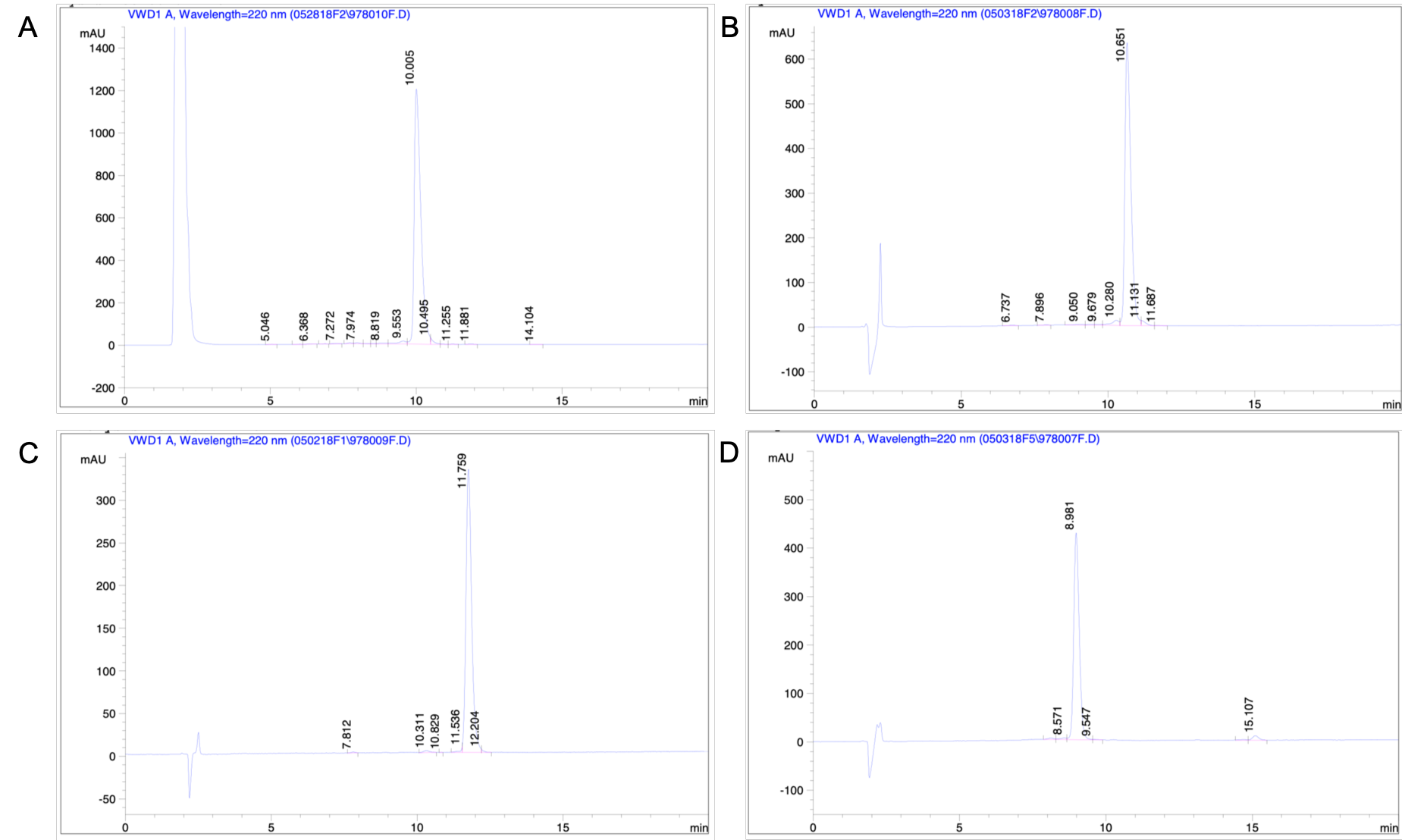

Supplement: Supplementary file 2 [file Image_2.tiff]

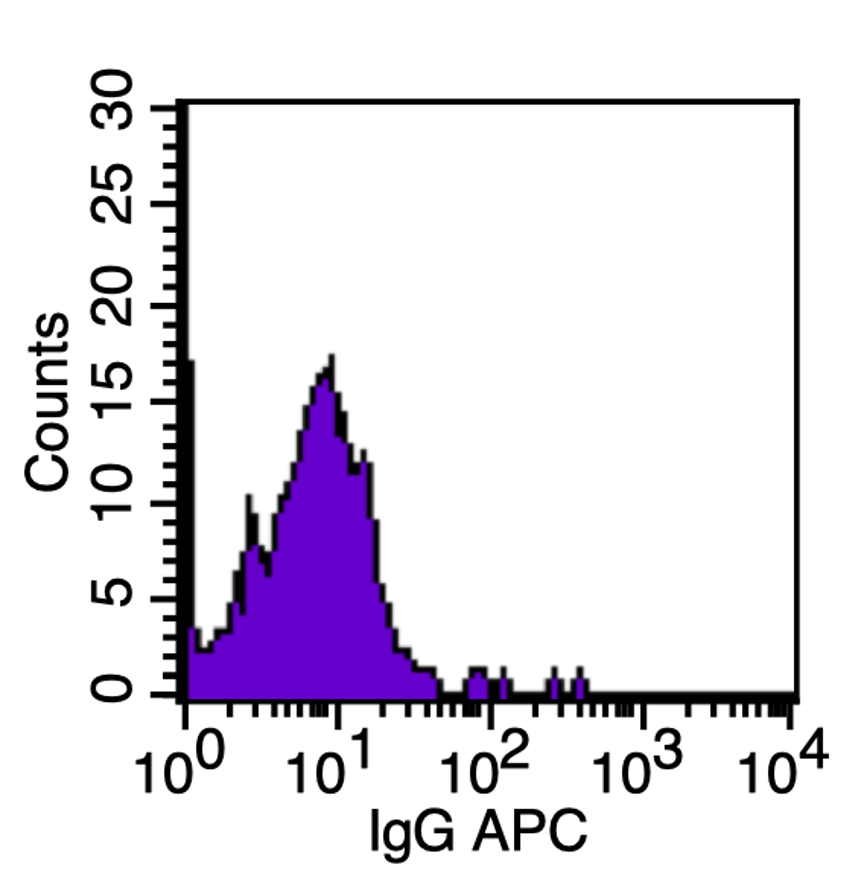

Supplement: Supplementary file 3 [file Image_3.png]

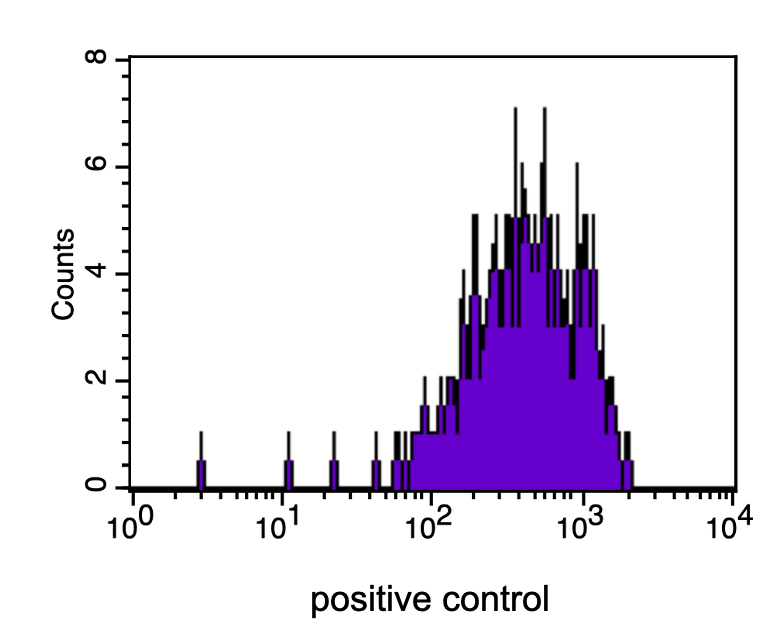

Supplement: Supplementary file 4 [file Image_4.png]
